# Nencki Affective Picture System: Cross-Cultural Study in Europe and Iran

**DOI:** 10.3389/fpsyg.2017.00274

**Published:** 2017-03-03

**Authors:** Monika Riegel, Abnoos Moslehi, Jarosław M. Michałowski, Łukasz Żurawski, Marko Horvat, Marek Wypych, Katarzyna Jednoróg, Artur Marchewka

**Affiliations:** ^1^Laboratory of Brain Imaging, Neurobiology Centre, Nencki Institute of Experimental Biology, Polish Academy of SciencesWarsaw, Poland; ^2^Faculty of Psychology, University of WarsawWarsaw, Poland; ^3^Department of Psychology, Faculty of Social Sciences and Design in Poznan, SWPS University of Social Sciences and HumanitiesPoznan, Poland; ^4^Laboratory of Psychophysiology, Department of Neurophysiology, Nencki Institute of Experimental Biology, Polish Academy of SciencesWarsaw, Poland; ^5^Department of Computer Science and Information Technology, University of Applied SciencesZagreb, Croatia

**Keywords:** cross-cultural comparison, affective visual stimuli, basic emotions, valence, arousal, Nencki Affective Picture System (NAPS)

## Abstract

Although emotions have been assumed conventionally to be universal, recent studies have suggested that various aspects of emotions may be mediated by cultural background. The purpose of our research was to test these contradictory views, in the case of the subjective evaluation of visual affective stimuli. We also sought to validate the recently introduced Nencki Affective Picture System (NAPS) database on a different cultural group. Since there has been, to date, no attempt to compare the emotions of a culturally distinct sample of Iranians with those of Europeans, subjective ratings were collected from 40 Iranians and 39 Europeans. Each cultural group was asked separately to provide normative affective ratings and classify pictures according to discrete emotions. The results were analyzed to identify cultural differences in the ratings of individual images. One hundred and seventy NAPS pictures were rated with regard to the intensity of the basic emotions (happiness, sadness, fear, surprise, anger, and disgust) they elicited, as well as in terms of affective dimensions (valence and arousal). Contrary to previous studies using the International Affective Picture System, our results for Europeans and Iranians show that neither the ratings for affective dimensions nor for basic emotions differed across cultural groups. In both cultural groups, the relationship between valence and arousal ratings could be best described by a classical boomerang-shaped function. However, the content of the pictures (animals, faces, landscapes, objects, or people) had a significant effect on the ratings for valence and arousal. These findings indicate that further studies in cross-cultural affective research should control for the content of stimuli.

## Introduction

It was Charles Darwin who first proposed that discrete emotions had a physiological basis, associated with facial signals that were universal (Darwin, 1872). However, according to numerous recent studies, various aspects of emotion, such as expression, perception, regulation, and recognition, may be culture dependent (Scherer and Wallbott, 1994; Elfenbein and Ambady, 2003; Jack et al., 2012; Lindquist et al., 2012). It has been proposed that cultural background is one of the key factors affecting emotion processing (Eid and Diener, 2001), as reflected in differences identified between cultural groups in behavioral and neuroimaging studies.

There are numerous ways in which emotions can be studied across cultures in laboratory experiments. One of the most popular is the use of affective images as stimuli, which has several advantages over alternative methods. The physical parameters of static emotional stimuli are easy to control, facilitating their selection, manipulation, and the interpretation of results. Standardized datasets have been developed, including faces and emotional scenes (e.g., Lang et al., 2008; Marchewka et al., 2014). Experiments using standardized affective pictures as stimuli have revealed cross-cultural differences in terms of the intensity with which emotions are expressed or experienced, and their neural correlates. For instance, compared to West European cultures, East Asian cultures discourage intense expressions of emotion, in order to maintain collective harmony (Matsumoto et al., 2005; Matsumoto and Fontaine, 2008). As a consequence, when viewing Ekman’s fearful faces, Japanese people have been found to respond with less intensity and with greater inhibitory activation of the right inferior frontal areas compared to Caucasians, who showed activation of classical networks associated with more intense emotional responses, including in the left amygdala (Moriguchi et al., 2005).

Affective ratings reveal the influence of culture also on experiential and behavioral responses to emotional stimuli (Mauss and Butler, 2010). Comparison of affective ratings across cultural groups is important to evaluate the generalizability of studies (Lohani et al., 2013).

### Emotion – Definitions

There have been several attempts in recent decades to categorize human affective experiences. The best known are the theories of discrete emotions and of affective dimensions. Discrete emotion theories hold that there are 6–10 core discrete emotions, which are universal (Ekman and Friesen, 1971). Happiness, sadness, anger, fear, surprise, and disgust have been proposed as the basic categories of emotion, triggered by specific antecedent events, and related to certain physiological reactions and facial expressions (Ekman, 1992). On the other hand, according to dimensional models of emotion inspired by Wundt (1897/1998), all emotional states may be arranged in a two- or three-dimensional space. The circumplex model, including the dimensions of valence and arousal, is one of the most influential in the field of affective neuroscience (Russell, 1980). In this model, valence refers to the positive vs. negative value of the emotional state, while arousal refers to the intensity of emotional arousal or excitement. Although there is a U-shaped relationship between these two dimensions, it has been recently proposed that they can be used to identify a wide range of variations at the individual level, such as personality or cultural correlates (Kuppens et al., 2013). As for cultural differences in the experience of basic emotions, contradictory results have been obtained depending on the modality of stimuli used, and there has been a longstanding debate over their significance. The recognition of non-verbal emotional vocalizations has been found to be universal (Sauter et al., 2010), while differences have been indicated in emotion responses to musical segments (Argstatter, 2015) and facial expressions (Jack et al., 2012; Gendron et al., 2014).

Given the inconsistent findings with regard to cultural differences related to affective dimensions and discrete emotions, a more holistic theoretical framework is needed, combining dimensional and discrete models (Russell, 2003; Briesemeister et al., 2014). However, a significant challenge before such a synthesis can be made is the choice of appropriate affective stimuli to evoke specific emotional states, including across cultures.

### Standardized Datasets of Affective Pictures

Only a few datasets of visual affective stimuli have been standardized according to both affective dimensions and discrete emotions (Bradley and Lang, 1994; Lang et al., 2008; Stevenson and James, 2008). The best known is probably the International Affective Picture System (IAPS; Lang et al., 2008). In the IAPS normalization study, participants were asked to view images one by one and rate the degree to which they felt aroused or calm, together with the pleasantness or unpleasantness of each image, using subjective Likert-type scales such as the Self-Assessment Manikin (SAM; Bradley and Lang, 1994). The mean valence and arousal ratings for each picture were plotted in a two-dimensional affective space. The unsymmetrical, boomerang-shaped plots revealed a statistical association between the level of arousal and valence. Pictures with very negative valence ratings induced greater arousal than very positively rated pictures. Neutral levels of valence were associated with lower levels of arousal (Lang et al., 2008). Subsequent studies performed using IAPS have shown that valence-arousal distribution is associated with the content of the pictorial stimuli. For example, human content images are located in the high arousal/positive and high arousal/negative areas of affective space, whereas inanimate objects are represented in the low arousal/neutral valence area (Colden et al., 2008).

The correlation between valence and arousal ratings shown by the IAPS normalization study has been replicated in different cultures (e.g., Verschuere et al., 2001; Ribeiro et al., 2005; Bradley and Lang, 2007; Deak et al., 2010; Dufey et al., 2011; Silva, 2011; Drace et al., 2013; Soares et al., 2015). However, the results of these cross-cultural studies have consistently indicated that the mean rating of arousal differs across cultures (Ribeiro et al., 2005; Bradley and Lang, 2007). Exceptions include normative studies conducted among Flemish, German, and Hungarian samples, in which the mean level of arousal did not differ from US norms (Verschuere et al., 2001; Deak et al., 2010). Other cultural groups, including Bosnians, Chileans, Italians, and Brazilians, reported higher levels of arousal in response to the IAPS pictures compared to US ratings, whereas Swedes indicated lower arousal (Bradley and Lang, 1994; Silva, 2011; Drace et al., 2013).

Notwithstanding its popularity, the IAPS dataset suffers from certain shortcomings (Mikels et al., 2005; Colden et al., 2008; Dan-Glauser and Scherer, 2011; Grabowska et al., 2011), including the limited number of pictures for each content category, the unsatisfactory quality of some photographs, and out-dated content. Recently, the Nencki Affective Picture System (NAPS; Marchewka et al., 2014) was introduced as an alternative standardized dataset of affective visual stimuli. The dataset contains only high quality images (minimum resolution 1,600 by 1,200 pixels), with such physical properties as luminance, contrast, and color composition. The dataset is divided into five content categories (animals, faces, people, landscapes, and objects), each of which has demonstrated a typical valence-arousal relationship in both males and females. The first ratings for NAPS pictures formed a more linear affective space than those for IAPS, most likely due to the slider scale used for the initial collection of normative ratings (ratings collected subsequently on the SAM scale formed a typical boomerang-shaped function). NAPS also contains norms for the intensity of basic emotions (e.g., sadness, happiness, anger, fear, surprise, and disgust) and the pictures have been categorized accordingly (see **Figure 2** with exemplary stimuli; Riegel et al., 2016). These advantages allow for more accurate selection of affective stimuli.

### Research Rationale

The present study aimed to provide a two-pronged extension of previous research. On one hand, we sought to validate the recently introduced NAPS database on a distinct, non-European cultural group. On the other hand, it was an initial attempt at studying emotion assessment among a sample of Iranians. As with other standardized tools used in affective research, NAPS requires to be validated in studies on various cultural groups. Most cross-cultural studies of emotion processing have compared Western European or North American with East Asian samples. Reported differences in the experience (Lim, 2016) and perception (Uono and Hietanen, 2015) of emotions, as well as between the related cognitive mechanisms (Engelmann and Pogosyan, 2013), have usually been explained with reference to factors such as different levels of economic development, and norms associated with religions or the individualism/collectivism divide (Hofstede, 2010). Our choice to compare Iranian and European (Polish, Croatian) samples was motivated by recognition of the distinct cultural characteristics of each group, as well as by the relative lack of studies on emotion assessment in Iranian culture. Iran is geographically situated in the Middle East, yet differs from other Asian cultures in terms of language, historical background, political system, ethnicity, and social norms. Nonetheless, it is representative of more collectivistic, less wealthy, Islamic cultures (Joshanloo and Bakhshi, 2015).

Recent studies have suggested that there may be differences between Iranian and Western cultures in terms of emotion processing. For instance, Iranian students were found to show more internalizing and externalizing symptoms than German students (Tahmouresi et al., 2014). In another study, the Iranian sample scored lower on the frequency of experiencing positive affect and higher on the frequency of experiencing negative affect, in comparison to an American sample (Joshanloo and Bakhshi, 2015). It has been proposed that Western individualistic cultures may promote more positive emotions than the more collectivistic and less wealthy Iran. In Iranian-Islamic culture, expressions of happiness may be censured, while the expression of negative emotions is sometimes praised and encouraged (Joshanloo and Weijers, 2014). The development of emotion regulation has been found to be suppressed among Iranian children, by the need to show respect and maintain harmony within families (Tahmouresi et al., 2014). It is therefore surprising that Iran is one of the least studied nations in terms of affective psychology. Our general hypothesis was that cultural differences would determine differences in emotion processing between Europe and Iran, which would be reflected in the ratings given to affective pictures.

For the purposes of our study, we incorporated theoretical models of both discrete emotion categories and affective dimensions. Based on previous research using IAPS, we hypothesized that NAPS ratings for valence and arousal would form a typical boomerang-shaped affective space in both cultures. However, we expected to observe cultural differences in the mean NAPS ratings and the ratings for individual images, in the affective dimensions and for ratings of basic emotions. We also aimed to explore the relationship between affective dimensions and discrete emotion categories in each culture. We expected that in both cultural groups this relationship would be heterogenous, meaning that the relationships between affective dimensions and basic emotions would vary from one emotion to another. The pictures would then be classified according to the specific basic emotions they elicited in each cultural group separately. Here, we expected to be able to distinguish fewer pictures related specifically to happiness based on the assessments of the Iranian sample than for the European sample. Finally, we attempted to investigate whether picture content categories and cultural group had an effect on the ratings for affective dimensions and basic emotions. We expected to find no cultural differences in the respect of content categories. However, we posited that regardless of content category, the mean level of arousal and happiness would be higher in Europe than in Iran. The aim was to enable researchers to select pictures from particular content categories and related to specific basic emotions, as well as to compare European and Iranian cultures in terms of certain affective dimensions and basic emotions.

## Materials and Methods

### Participants

A group of 40 students, including 17 men (*M*_age_ = 25.12, *SD* = 3.37) and 23 women (*M*_age_ = 26.30, *SD* = 3.63), between the ages of 18 and 35, were recruited at universities in Tehran, Iran, via social media, advertisements placed in a library, and snowball sampling. The criteria for participation were proficiency in English and age group. Data collected from the Iranian sample was compared to that collected from a sample of 39 students of various European nationalities (Polish – 30, Croatian – 6, Spanish – 2, French – 1), studying in Warsaw. This sample consisted of 24 women (*M*_age_ = 23.54, *SD* = 4.83) and 15 men (*M*_age_ = 23.33, *SD* = 4.67) between the ages of 18 and 35. Although the participants were of different nationalities, Europeans have been treated as a homogenous group in previous cross-cultural affective studies (Gregory and Varney, 1996; Sauter et al., 2010; Ferdenzi et al., 2013). The European sample of students included participants from a NAPS follow-up study, which had aimed to provide norms for basic emotions (Riegel et al., 2016). The European group (*M*_age_ = 23.46, *SD* = 4.71) was younger overall than the Iranian group (*M*_age_ = 25.8, *SD* = 3.53), *t*(77) = 2.5, *p* = 0.015, *d* = 0.57. However, both groups comprised only students in the same age range. None of the participants who took part in the study declared a history of neurological illness of any sort, nor was undergoing treatment with psychoactive drugs.

### Materials

The NAPS dataset consists of 1356 realistic photographs, either taken by the co-authors in locations around the world in the years 2006–2012 or obtained from the non-commercial photography stock of a Polish newspaper group. Only pictures that do not contain any visible commercial logotypes, well-known locations, or culture-specific items are included in the dataset. The pictures are sorted into five content categories. In this study, we used a set of 170 images from the subset of the NAPS database with normative ratings for basic emotions (NAPS BE; Riegel et al., 2016). Based on reports using IAPS (IAPS; Bradley and Lang, 2007), which showed that the distribution of stimuli across the valence and arousal dimensions is related to human vs. inanimate picture content (Colden et al., 2008), in order to cover the whole affective space we chose and counterbalanced pictures from each content category. The selection of images reflected our desires to: (1) avoid repetitive stimuli patterns; (2) maintain content variety in all the discrete emotion categories; (3) limit the number of neutral stimuli and (4) cover the whole affective space. We used 53 images of faces, 34 of objects, 33 of animals, 33 of people, and 17 of landscapes. These proportions are representative of the proportions of the NAPS BE dataset as a whole (Riegel et al., 2016). The images of landscapes were less arousing and of neutral valence (see **Table 2**; Marchewka et al., 2014). A detailed list of the images used in this study can be found in the Supplementary Table S1. The set was divided into four sub-sets for the purpose of randomization. The NAPS characteristics of the subset in terms of *M (SD)* are as follows: happiness 2.46 (1.29); sadness 2.04 (1.29); fear 1.77 (0.79); surprise 1.87 (0.65); anger 1.52 (0.74); disgust 1.70 (0.96); arousal 3.40 (1.06); valence 4.87 (1.58).

### Procedure

**FIGURE 1:**
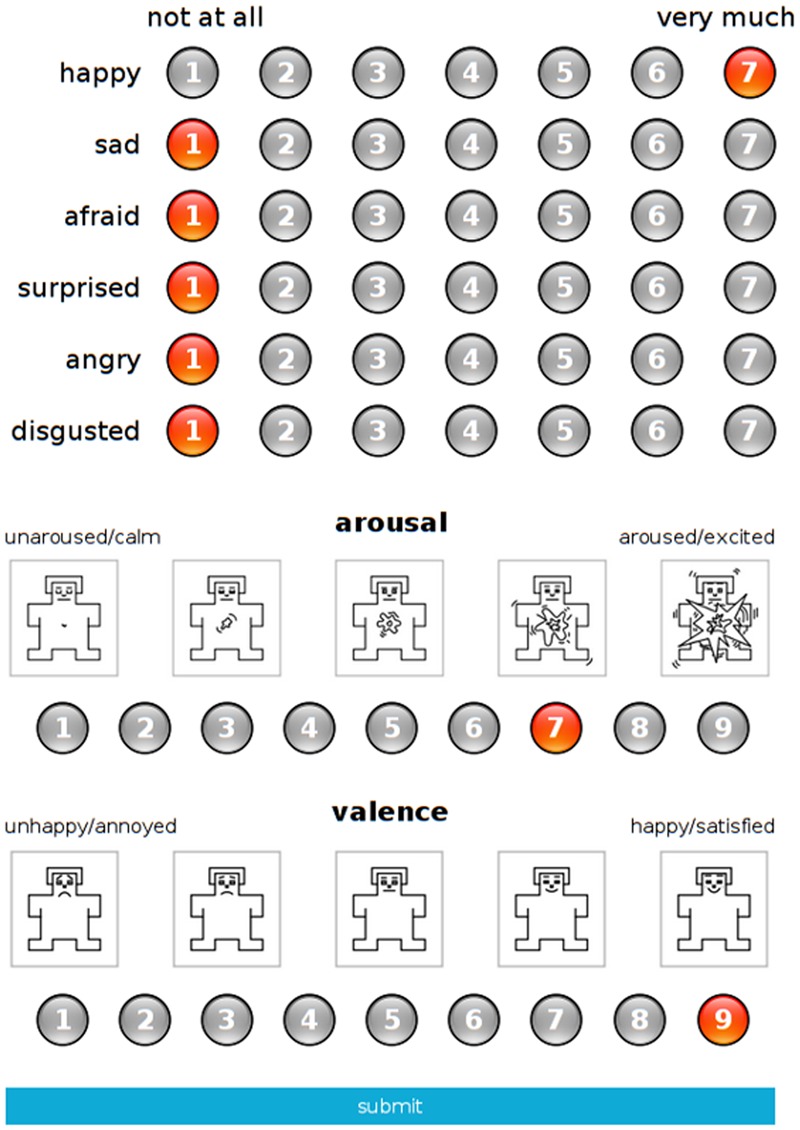
**Rating scales on the web-based online platform developed for the study**.

Experimental sessions were carried out in the Nencki Institute of Experimental Biology in Warsaw and in the Azimi library in Teheran. The procedure was conducted in English, identically for both cultural groups. All technical conditions were comparable, as controlled by the same monitoring person (AMo). First, each participant signed an informed consent document. Exemplary snapshots of the platform (**Figure 1**) and of the Self-Assessment Manikin scales (Bradley and Lang, 1994) were then presented to the participants, with oral instructions adapted from Lang et al. (2008). Once their demographic details had been collected, the participants were given brief written instructions similar to those that had been presented orally. At both locations, participants completed their tasks individually, in silent rooms. The intensity of discrete emotions was rated on a 7-point Likert scale (1 = not at all, 7 = very much), while affective dimensions were rated using a modified 9-point Likert scale of SAM (for arousal: 1 = unaroused/calm, 9 = aroused/excited; for valence: 1 = unhappy/annoyed, 9 = happy/satisfied). These rating scales were adopted from previous IAPS and NAPS BE studies (Mikels et al., 2005; Stevenson et al., 2007; Lang et al., 2008; Riegel et al., 2016). In total, eight ratings were collected for each image from each participant. Each rating session lasted around 40–60 min (depending on the speed with which the individuals assessed their responses). Upon finishing the task, each participant received a payment of 5 EUR. The study protocol was approved by the ethics committee at the Experimental Research, Faculty of Psychology, University of Warsaw, both for data collection in Warsaw and in Teheran.

**FIGURE 2:**
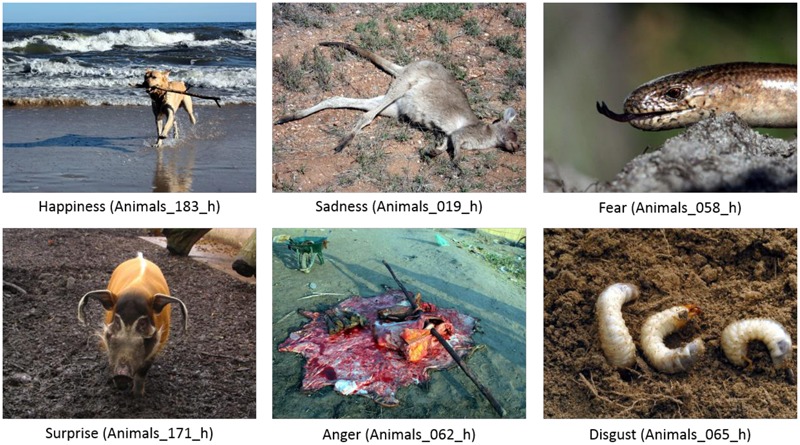
**Sample of standardized images classified as representing each basic emotion in the semantic category of animals**.

The ratings were collected through an online platform created for the purpose of the study. Images from each category were presented pseudorandomly, with no more than three images from the same content category shown consecutively. **Figure 2** provides an exemplary sample of images for each basic emotion in the category of animals. In order to avoid serial position (primacy and recency) effects, each subset of 170 pictures was divided into three parts. These were positioned in one of three possible ways and were counterbalanced across the participants. Each image was presented in the center of the screen for 3 s, after which it was moved to the left side of the screen. On the right side, separate scales were presented for six discrete emotion categories (happiness, sadness, anger, fear, disgust, and surprise) and affective dimensions (valence, arousal). The PC displays were positioned at a distance of 60 cm in front of each participant.

### Data Analysis

Having calculated the means and standard deviations of the ratings on each of the eight scales for all pictures, we removed the individual outlier ratings, defined as individual ratings ±3 standard deviations from the means for both samples. As for single images, we removed 1–3 individual ratings per image (2,5–7,5%) among Iranians and 1–3 individual ratings per image (2,5–7,6%) among Europeans. Thus, no image was fully removed from the analyses as an outlier. This approach to outlier removal was based on the previous studies on emotion processing (Righart and de Gelder, 2008; Dan-Glauser and Scherer, 2011; Brouwer et al., 2013; Lakens et al., 2013) and in line with previous analyses on the NAPS affective ratings (Marchewka et al., 2014; Riegel et al., 2016).

Data analysis was then performed in several stages. In order to investigate the relationship between the affective dimensions in each cultural group, we divided the pictures into negative and positive classes (according to their mean valence ratings), and then compared the correlation coefficients for valence and arousal, transforming them into *z*-scores. Regression analysis was then performed, to explore whether the ratings for affective dimensions obtained formed a typical boomerang-shaped affective space. To identify single pictures rated differently across cultures, we compared the mean ratings given by the Iranian and European samples on each affective scale separately. The significance of differences in the ratings was calculated using the Mann–Whitney–Wilcoxon *U* test (MWW). To compare the relationships between affective dimensions and basic emotions in each sample, further regression analyses were performed using the categorical data for each picture to predict the dimensional data. Partial correlations were also calculated. Pictures eliciting one basic emotion significantly more than others were identified, and each picture was subsequently classified as belonging to a particular discrete emotion category for one of the two cultural groups. This classification method was based on the mean subjective ratings given by the participants and with 85% non-overlapping confidence intervals. In the next step of data analysis, we performed analysis of variance (ANOVA), in order to compare the mean ratings of affective dimensions (the dependent variable) across cultural groups (the between-subject factor) and picture content categories (the within-subject factor). Finally, we performed a similar analysis of variance in order to compare the mean ratings of discrete emotions across cultural groups and picture content categories. The data analysis described above should encourage future users of NAPS to use all of the provided norms and variables in cross-cultural studies.

## Results

### Ratings for Affective Dimensions

The relationships were examined between the ratings for valence (Iran *M* = 4.71, *SD* = 1.35; Europe *M* = 4.87, *SD* = 1.58) and arousal (Iran *M* = 3.41, *SD* = 1.06; Europe *M* = 3.40, *SD* = 1.06) in each sample. According to criteria described in previous studies (Kissler et al., 2007; Ferré et al., 2012), the pictures were divided into negative and positive categories, based on a mean cut-off valence rating of 5.00. The Iranians rated 78 pictures as positive and 91 as negative. The Europeans evaluated 84 pictures as positive and 85 as negative. In each sample, one image (for Iranians, of an elderly woman, for Europeans of an alligator) received a mean rating of 5.0 in the valence dimension and was not included in the classification. Taking images as cases, Pearson’s correlations analysis revealed a strong positive correlation between arousal and valence in pictures evaluated as positive, among both Iranians (*r* = 0.43, *p* < 0.001) and Europeans (*r* = 0.74, *p* < 0.001). A significant negative correlation was observed between arousal and valence in pictures evaluated as negative by both Iranians (*r* = -0.76, *p* < 0.001) and Europeans (*r* = -0.85, *p* < 0.001). This means that the more positively or negatively a picture was evaluated, the more arousing it was it was also rated as being (see **Table 1**).

**Table 1:** Correlation coefficients calculated for arousal and negative/positive valence for each cultural sample.

	Valence > 5.0		Valence < 5.0	
Group	Arousal	*N*	Arousal	*n*
Iran	0.43^∗∗^	78	-0.76^∗∗^	91
Europe	0.74^∗∗^	84	-0.85^∗∗^	85

*^∗∗^p < 0.001. Images with mean ratings above or below 5.0 on the valence scale were dichotomized into positive and negative categories before separate correlation analysis.*

To enable direct comparison, similar to previous studies (Marchewka et al., 2014; Riegel et al., 2016), we used Fisher’s *r*-to-*z* transformation to calculate the correlation coefficients for Iranian and European experimental groups as *z*-scores (Preacher, 2002). Taking into account the sample size, the *z*-scores were compared using formula 2.8.5 from Cohen and Cohen (1983), i.e., Z1 - Z2 = SDZ, where SDZ = Sqrt [1/(N1 - 3) + 1/(N2 - 3)] and N1 and N2 are the sample sizes. Comparison of the correlation coefficients for each culture revealed a significant difference only for positive images (*z* = -3.1, *p* = 0.002). For positive images, valence and arousal were much less strongly correlated in the Iranian sample.

**FIGURE 3:**
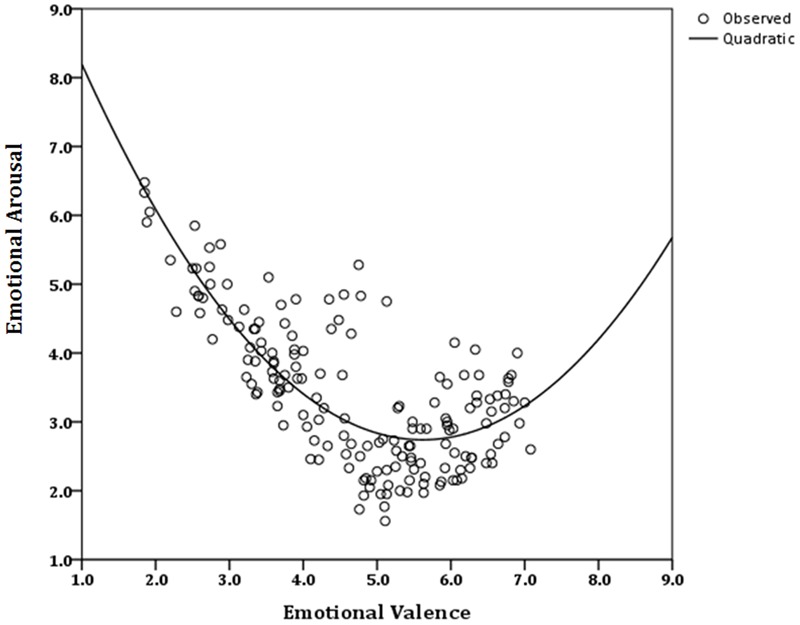
**Quadratic function fitting arousal to the whole range of valence, for ratings of NAPS pictures by the Iranian group**.

**FIGURE 4:**
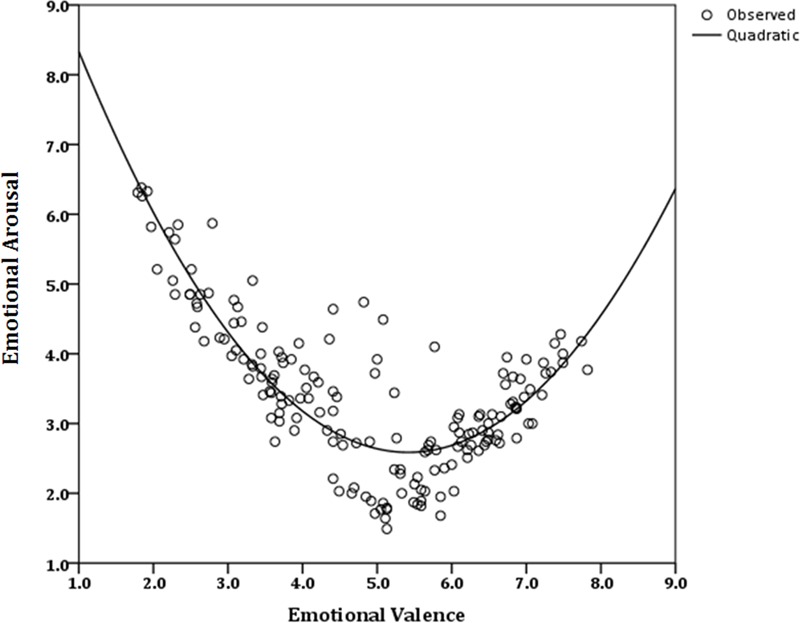
**Quadratic function fitting arousal to the whole range of valence, for ratings of NAPS pictures by the European group**.

Since our hypothesis was confirmed and the distribution of ratings in affective space was similar to the classical boomerang shape (Lang et al., 2008), we conducted a regression analysis to further investigate the relationship between valence and arousal in each sample (Ferré et al., 2012; Monnier and Syssau, 2014; Montefinese et al., 2014; Hinojosa et al., 2015). A quadratic function best described the relationship between valence and arousal in the Iranian sample [*y* = 10.81 + (-2.88)*x* + 0.26, R2 = 0.63]. Valence accounted for 63% of variance in the arousal ratings (**Figure 3**). Valence was also a significant predictor of arousal ratings among Europeans [*y* = 11.23 + (-3.19)*x* + 0.29, R2 = 0.74]. Variance of arousal ratings were predicted with 74% accuracy by the valence ratings (**Figure 4**).

### Cross-Cultural Differences between Ratings for Images

**FIGURE 5:**
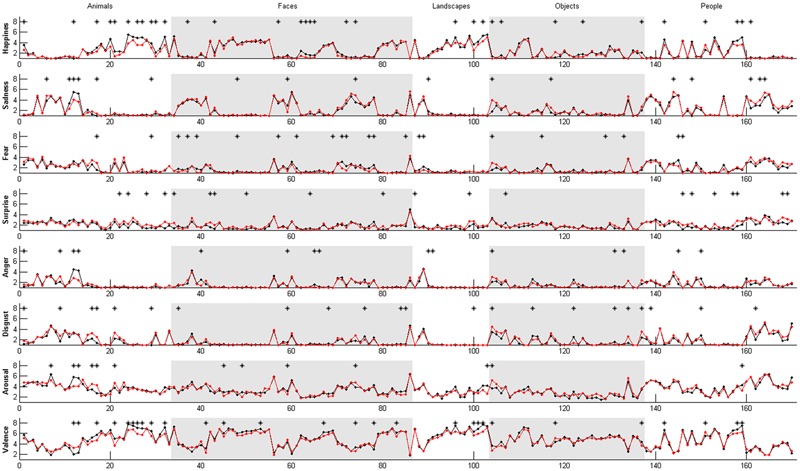
**Distribution of mean ratings at each affective scale for each picture compared across both groups. black – Europe, red – Iran; ^∗^indicates significant cross-cultural differences in mean ratings at *p* < 0.05**.

In order to gain clearer insight into cross-cultural similarities and differences between the Iranian and European samples, we compared the ratings given on each scale (happiness, sadness, anger, fear, disgust, surprise, arousal, and valance) to each picture separately (Wierzba et al., 2015). The significance of differences between the ratings was calculated using the Mann–Whitney–Wilcoxon *U* test (MWW) which unlike *t*-tests, does not require an assumption of normal distribution. In line with our second hypothesis, we observed differences in the ratings for individual images. The results are presented in **Figure 5**, where asterisks indicate significant cross-cultural differences in mean ratings of *p* < 0.05. The full list of images, showing exact differences in mean ratings across the two groups for each picture separately, are presented in Supplementary Table S2.

### Relationship between Discrete Emotions and Affective Dimensions

The relationships between discrete emotion categories and affective dimensions were explored in each group (Europeans and Iranians). In line with previous studies, four separate regression analyses were conducted, to assess to what extent the intensities of six discrete emotion categories predicted the mean ratings for valence and arousal in the cases of negatively (rating <5) or positively rated pictures (rating >5) (Ric et al., 2013; Riegel et al., 2016). A regression analysis of the Iranian sample revealed that, for both positively and negatively rated pictures, discrete emotion categories significantly predicted mean ratings for valence. For positive images, *F*(6,68) = 106.94, *p* < 0.001, *R*^2^ = 0.90, while for negative images *F*(6,87) = 188.95, *p* < 0.001, *R*^2^ = 0.92. Similarly, discrete emotion categories explained the variance in mean ratings for arousal in response to both positive [*F*(6,68) = 35.24, *p* < 0.001, *R*^2^ = 0.76] and negative images [*F*(6,87) = 182.89, *p* < 0.001, *R*^2^ = 0.93]. The results of regression analysis are presented in **Tables 2, 3**.

**Table 2:** Regression analysis and partial correlations showing discrete emotion category ratings predicting valence for negative/positive images in each cultural group separately.

Valence class/Emotion	Europeans			Iranians		
	β	*t*	Partial *r*	β	*t*	Partial *r*
**Negative pictures**						
Happiness	0.16	3.30**	0.35	0.17	4.34***	0.42
Sadness	-0.59	-9.24***	-0.72	-0.64	-12.56***	-0.80
Fear	-0.20	-3.60**	-0.38	-0.17	-3.08**	-0.31
Surprise	0.03	0.41	0.05	0.04	0.62	0.07
Anger	-0.05	-1.09	-0.12	-0.03	-0.74	-0.08
Disgust	-0.30	-5.82***	-0.55	-0.24	-5.46***	-0.50
**Positive pictures**						
Happiness	0.86	18.96***	0.90	0.80	17.51***	0.90
Sadness	-0.001	-0.02	-0.002	-0.12	-2.60*	-0.30
Fear	0.04	0.71	0.08	-0.08	-1.32	-0.16
Surprise	0.04	0.82	0.09	0.20	4.30***	0.46
Anger	-0.10	-2.05*	-0.22	-0.11	-1.72	-0.20
Disgust	-0.10	-2.37*	-0.26	0.01	0.26	0.03

*^∗^*p* < 0.05, ^∗∗^*p* < 0.01, ^∗∗∗^*p* < 0.001.*

**Table 3:** Regression analysis and partial correlations showing discrete emotion category ratings predicting arousal for negative/positive images in each cultural group separately.

Valence class/Emotion	Europeans			Iranians		
	β	*T*	Partial *r*	β	*T*	Partial *r*
**Negative pictures**						
Happiness	0.13	2.61*	0.28	0.09	2.23*	0.23
Sadness	0.40	5.89***	0.56	0.42	7.98***	0.65
Fear	0.49	8.43***	0.69	0.63	10.94***	0.76
Surprise	0.09	1.35	0.15	0.04	0.63	0.07
Anger	0.03	0.67	0.08	0.08	1.74	0.18
Disgust	0.31	5.70***	0.54	0.08	1.73	0.18
**Positive pictures**						
Happiness	0.85	14.87***	0.86	0.36	4.91***	0.51
Sadness	0.12	2.18*	0.24	0.01	0.14	0.02
Fear	0.39	6.15***	0.57	0.34	3.43**	0.38
Surprise	0.05	0.78	0.09	0.40	5.36***	0.54
Anger	0.09	1.49	0.16	0.08	0.78	0.09
Disgust	0.13	2.40*	0.26	0.22	2.98**	0.34

*^∗^p < 0.05, ^∗∗^*p* < 0.01, ^∗∗∗^*p* < 0.001.*

In the European sample, regression analysis showed that discrete emotion categories significantly predicted mean valence ratings in the case of both positive images [*F*(6,79) = 103.39, *p* < 0.001, *R*^2^ = 0.89] and negative images [*F*(6,77) = 119.57, *p* < 0.001, *R*^2^ = 0.90]. Discrete emotion categories also accounted for variances in the mean arousal ratings. For positive images, *F*(6,79) = 61.17, *p* < 0.001, *R*^2^ = 0.82, and for negative images *F*(6,76) = 105.46, *p* < 0.001, *R*^2^ = 0.89. The results of regression analysis are presented in **Tables 2, 3**.

To obtain a deeper understanding of these relationships, we performed partial correlation analyses on both samples. As with the original NAPS ratings (Riegel et al., 2016), partial correlations (representing the unique influence of one predictor relative to the part of the variance of a dependent variable unexplained by the other predictors) revealed that basic emotions contributed to valence and arousal in different ways (Ric et al., 2013), which confirmed our hypothesis that in both cultural groups this relationship would be heterogenous.

In the case of positive pictures, among Iranians, arousal was related to happiness, fear, surprise, and disgust. In response to pictures evaluated positively by the European sample, arousal was related to happiness, fear, disgust, and sadness. As far as valence is concerned, pictures rated as being positive were related to happiness, sadness, and surprise for Iranians, while in the group of Europeans positive valence was related to happiness, anger, and disgust. Neither sample showed anger related to arousal, nor was fear related to positive valence in either of the two samples.

In the case of pictures rated negative, arousal was related to happiness, sadness, and fear by both Iranian and European samples, as well as to disgust in the European group. Among pictures rated as being negative, valence was related to happiness, sadness, fear, and disgust in both the Iranian and European samples. Anger and surprise were related neither to negative valence nor arousal. The results of partial correlation analysis are presented in **Tables 2, 3**.

### Picture Classification to Discrete Emotions

According to the procedure proposed by Mikels et al. (2005) an used in other studies (Stevenson et al., 2007; Riegel et al., 2016), the images were classified into discrete emotion categories for each cultural group, on the basis of an 85% confidence interval overlap between discrete emotion ratings. This method of classification allows pure, blurred, and undifferentiated emotional categories to be differentiated, based on subjective emotion ratings for each photograph (happiness, surprise, fear, sadness, disgust, and anger). An image was sorted into one of the pure discrete emotion categories if its mean rating for a particular discrete emotion category was higher than those for other categories, and if the confidence intervals (CI) did not overlap with those for other categories. A picture was qualified as blended when two or three mean ratings for discrete emotion categories were higher than those for others, and when the CIs also overlapped. Images were considered undifferentiated when more than three mean ratings for discrete emotion categories were higher than those for other categories.

Of the 170 pictures rated by Iranians, 102 (60%) were classified as pure emotion categories, namely: happiness = 65 (63.7%), sadness = 29 (28.4%), disgust = 4 (3.9%), and fear = 4 (3.9%). Based on the ratings given by the Europeans, 116 (68.2%) of the total 170 pictures were classified into pure emotion categories with the following frequency: happiness = 76 (65.5%), sadness = 26 (22.4%), disgust = 10 (8.6%), fear = 4 (3.4%). These results confirmed our hypothesis – we were able to distinguish fewer pictures related specifically to happiness based on the assessments of the Iranian sample than for the European sample. There were no images classified as pure emotions of surprise or anger in any of the samples, as their CIs overlapped with one or more of the other emotion categories.

### Affective Dimensions across Cultures and Content Categories

We calculated the mean ratings by individual participants of pictures representing each content category for valence and arousal. Based on our previous study (Wierzba et al., 2015), we used the mean valence and arousal ratings separately as dependent variables, and performed ANOVAs with cultural group as a between-subject factor (two levels: Iranian, European) and using content category as a within-subject factor (five levels: faces, landscapes, people, objects, and animals). Since we analyzed the mean category ratings, the normality assumption was satisfied by the laws of the central limit theorem (Field, 2013).

The results for arousal revealed the that picture content category was the main determining factor: *F*(4,308) = 93.696, *p* < 0.001, η^2^ = 0.549 (see Appendix 1 for the results of *post hoc* pairwise comparisons, Bonferroni correction applied). Cultural group did not show a significant effect, against our hypothesis about cultural differences in the level of arousal: *F*(1,77) = 0.003, *p* < 0.956, η^2^ = 0.000. There was no interaction between the cultural group and the picture content category: *F*(1,77) = 0.003, *p* < 0.956, η^2^ = 0.000.

Similar analysis conducted for valence revealed the principal factor to be picture content category: *F*(4,308) = 170.373, *p* < 0.001, η^2^ = 0.689 (see Appendix 1 for the results of *post hoc* pairwise comparisons, Bonferroni correction applied). Again, cultural group did not appear to have a significant effect: *F*(1,77) = 3.087, *p* < 0.083, η^2^ = 0.039. Nor was there significant interaction between the cultural group and the picture content category: *F*(4,308) = 1.689, *p* < 0.177, η^2^ = 0.021.

Overall, no significant differences between the cultural groups were noted in terms of the mean ratings for affective dimensions, in accordance with our hypothesis. Descriptive statistics for each picture category are presented in **Table 4**.

**Table 4:** Descriptive statistics, calculated for each cultural group, for each affective dimension and picture content category separately.

Group/population	Faces (*n* = 53)		Landscapes (*n* = 17)		People (*n* = 33)		Objects (*n* = 34)		Animals(*n* = 33)	
	*M*	*SD*	*M*	*SD*	*M*	*SD*	*M*	*SD*	*M*	*SD*
**Iran**										
Valence	4.80	1.45	5.68	1.20	4.14	1.50	4.68	0.93	4.64	1.23
Arousal	3.25	1.00	2.87	0.64	4.07	1.15	2.9	0.90	3.86	0.86
**Europe**										
Valence	4.93	1.58	6.03	1.42	4.20	1.71	4.80	1.01	4.92	1.70
Arousal	3.31	0.92	3.10	0.86	4.06	1.16	2.66	0.84	3.84	0.93

*All scales ranged from 1 to 9, where 1 signifies negative/unarousing and 9 represents positive/arousing, for valence and arousal, respectively.*

### Discrete Emotions across Cultures and Content Categories

The mean ratings of pictures representing each content category were also calculated for individual participants, in terms of six discrete emotion categories. Using the mean ratings, we performed separate ANOVAs with each discrete emotion category as a dependent variable, cultural group as a between-subject factor (two levels: Iranian and European), and content category as a within-subject factor (five levels: faces, landscapes, people, objects, and animals), as in previous studies (Riegel et al., 2016). The results revealed that the primary factor was picture content category for all discrete emotion categories: happiness, *F*(4,308) = 141.607, *p* < 0.001, η^2^ = 0.648; sadness, *F*(4,308) = 160.355, *p* < 0.001, η^2^ = 0.676; fear, *F*(4,308) = 82.164, *p* < 0.001, η^2^ = 0.516; disgust, *F*(4,308) = 93.059, *p* < 0.001, η^2^ = 0.547; anger, *F*(4,308) = 25.095, *p* < 0.001, η^2^ = 0.246; surprise, *F*(4,308) = 41.011, *p* < 0.001, η^2^ = 0.348 (see Appendix 1 for the results of *post hoc* pairwise comparisons, Bonferroni correction applied). Neither the cultural group nor the interaction of cultural group and picture content category had a significant effect on the intensities of response reported for discrete emotion categories. In line with our hypothesis, we did not find cultural differences in the respect of content categories. However, we did not find a confirmation of our hypothesis about cultural differences in the level of happiness. Descriptive statistics for each discrete emotion category are presented in **Table 5**.

**Table 5:** Descriptive statistics for each discrete emotion category associated with each picture content category calculated separately.

Emotions/Group	Animals		Faces		Landscapes		Objects		People	
	*M*	*SD*	*M*	*SD*	*M*	*SD*	*M*	*SD*	*M*	*SD*
**Happiness**										
Europe	2.62	1.48	2.61	1.37	3.47	1.45	1.97	0.97	2.05	1.41
Iran	2.18	1.07	2.45	1.44	3.18	1.25	1.87	0.91	1.88	1.18
**Sadness**										
Europe	1.96	1.44	2.22	1.37	1.44	0.85	1.62	0.70	2.60	1.42
Iran	1.93	1.31	2.35	1.48	1.41	0.99	1.82	0.92	2.86	1.51
**Fear**										
Europe	2.04	0.85	1.66	0.72	1.28	0.30	1.46	0.57	2.25	0.89
Iran	2.25	0.98	1.49	0.71	1.19	0.15	1.47	0.68	2.27	0.93
**Surprise**										
Europe	2.10	0.58	1.72	0.69	1.53	0.31	1.78	0.51	2.16	0.74
Iran	2.31	0.50	1.79	0.68	1.77	0.47	1.80	0.47	1.80	0.54
**Anger**										
Europe	1.62	0.98	1.52	0.73	1.32	0.88	1.40	0.48	1.65	0.62
Iran	1.61	0.74	1.50	0.71	1.33	0.93	1.41	0.56	1.72	0.72
**Disgust**										
Europe	1.93	1.01	1.43	0.74	1.30	0.81	1.65	0.80	2.13	1.24
Iran	2.23	1.08	1.41	0.75	1.26	0.66	1.77	1.01	2.28	1.27

## Discussion

In this study, for the first time, the NAPS was used to directly compare affective ratings collected from two culturally different samples. The results are generally congruent with previous cross-cultural studies using the IAPS and the Self-Assessment Manikin. Regression analysis of the valence-arousal distribution indicates that, in both samples, pictures from NAPS elicit the highest levels of arousal when associated with the lower and upper ends of the valence scale. This relationship is best described by a quadratic function and represented by a boomerang-shaped distribution of affective space. The strong correlation between the affective dimensions in each group reveals that NAPS pictures elicited similar emotions across cultures.

Both groups rated negative pictures as being more arousing than positive pictures. This tendency to react more strongly toward negative stimuli is known as negativity bias, and is in line with previous findings concerning the motivation to approach or withdraw (Ito et al., 1998; Cacioppo and Gardner, 1999; Smith et al., 2003). It has been suggested that individual differences and the intensity of perceived negativity when rating negative stimuli can contribute to varying levels of negativity bias (Ito and Cacioppo, 2005; Yuan et al., 2007). In our study, the Iranian group rated more pictures negatively than did the European group. However, negativity bias was equally pronounced in both cultural groups. Although the cultural differences between individualistic Europeans and more collectivist Iranians are much stronger, this is in line with the findings of a cross-cultural comparison of Flemish and US ratings using IAPS pictures (Verschuere et al., 2001).

The results of our study do not support the hypothesis that arousal is a culture-sensitive component of affective states. They run counter to behavioral and psychological evidence, suggesting that more collectivist cultures tend to experience lower levels of arousal, possibly as a result of cultural norms (Berry et al., 2002; Moriguchi et al., 2005). For instance, Japanese subjects have been reported to experience lower levels of emotional arousal when viewing emotional pictures compared to French, as revealed by the later components of event-related potentials (ERPs), in a range from 255 to 455 ms in PCA (Hot et al., 2006). There is growing evidence showing the mediating role of culture on attentional biases during early visual affective perceptions (Blais et al., 2008; Jack et al., 2009, 2012). However, the results of our study of arousal did not reflect similar differences. It may be that Western influences on the demographic group used for the Iranian sample meant that their cultural norms, in terms of emotional responses, did not differ significantly from the European sample. Matsumoto and Yoo (2006) note that a large number of cross-cultural studies draw their samples from university students. This well-known sampling bias leads to a certain level of education equivalence in cross-cultural comparisons, which, together with the role of mass media, might explain some of the observed similarities across cultures.

In order to observe more detailed cross-cultural variations, which could have practical implications, we performed an MWW analysis for each of the images separately in terms of their ratings from each group on the different scales. We were thereby able to identify differences between particular images in the related affective dimensions (valence, arousal) and in terms of the basic emotions they aroused (happiness, sadness, anger, disgust, and fear), as well as images that were rated similarly across groups, depending on the research question.

In addition to the dimensional view, we collected normative ratings for discrete emotion categories. To the best of our knowledge, there have been no previous studies which have investigated cultural differences using affective visual stimuli within the theoretical framework of discrete emotion categories. Our results from regression analysis support previous findings, showing that the type of discrete emotion elicited by pictorial stimuli may be related to the levels of arousal and valence experienced by individuals (for a review see Lindquist and Barrett, 2008). We observed an intercorrelation between all discrete emotion categories (except anger), in terms of the mean ratings for arousal and valence given to both positive and negative images, across both cultural groups. Anger, being a more complex emotion generally, involves high levels of arousal, but contextually can be accompanied by sadness, disgust, or fear (Lindquist et al., 2012). Moreover, people tend to suppress undesirable reactive emotions, in order to avoid behavioral expressions of emotions that are socially disengaging, such as pride or anger (see e.g., Eid and Diener, 2001; Kitayama et al., 2006).

Partial correlations noted between the affective dimensions and basic emotions recorded in each cultural sample revealed a lack of homogeneity in terms of the ability of categorical ratings to predict dimensional ratings. In other words, categorical ratings cannot be extrapolated from dimensional ratings. This confirms the importance of combining both dimensional and categorical approaches in research on emotion (Stevenson et al., 2007), in order to design more ecologically valid paradigms.

Using a method adapted from Mikels et al. (2005), we categorized the NAPS pictures into discrete emotion categories based on 85% CI. We identified images belonging to single emotion categories, without overlaps between their mean ratings and those of other discrete emotions. In both cultural groups, NAPS photographs qualifying for inclusion in a single pure discrete emotion category were classified in the following order of frequency: happiness, sadness, disgust, and fear. Discrete emotions of anger and surprise were not rated as distinct emotions by either cultural group, which may be due to the small number of images rated by the participants. In our previous study, which provided norms for basic emotions elicited by a subset of the same NAPS dataset (NAPS BE; Riegel et al., 2016), anger and surprise were rated as distinct emotions. Several neuroimaging studies have attempted to investigate different neural pathways associated with discrete emotion categories, although their findings to date have been controversial (Vytal and Hamann, 2010; Lindquist et al., 2012). Nevertheless, the cross-cultural comparison of NAPS ratings described in this paper demonstrates its consistency and usefulness for investigating basic emotions in culturally distant groups, such as Iran.

The results of separate analysis of variance for each discrete emotion category reveal that, in both groups, the content of pictures contributed to the subjective ratings for happiness, sadness, fear, disgust, anger, and surprise. A significant correlation emerged between valence and arousal ratings in both groups for pictures belonging to all content categories except landscapes. This may be due to the smaller number of pictures in this content category (*n* = 17). Pictures in the landscapes category were also among the least arousing, which is in line with a generally observed negativity bias. Pictures of landscapes obtained the highest scores on the happiness scale. These pictures may also have lower affective properties in general, compared with faces or people (Marchewka et al., 2014). There were no highly arousing positive pictures in the NAPS subset used (NAPS ERO; Wierzba et al., 2015). Sadness, typically considered low in arousal (Wang et al., 2005), was elicited predominantly by the images of people. Stimuli related to people (e.g., faces) are typically used in studies concerning sadness (Schneider et al., 2000; Posse et al., 2003; Fitzgerald et al., 2004; Habel et al., 2005). Since this emotion is related to goal failure (Lench and Levine, 2008), it may be elicited less strongly by other types of stimuli, such as objects. Anger was the emotion elicited least strongly by the subset of NAPS stimuli used in our study, in both Iranian and European samples.

That picture content category had a significant effect on the ratings for arousal, valence, and discrete emotion categories highlights the importance of selecting proper stimuli in affective research. Cultural variations in this respect may stem from geopolitical differences or recent events. For instance, a cross-cultural comparison between Ghanaians and Americans demonstrated that Ghanaians showed more sensitivity to disgust and contamination. This may have been a survival-avoidance response to long-term infectious epidemics in Ghana (Skolnick and Dzokoto, 2013). Thus, differences observed in the affective responses of distinct cultural groups, for example regions with different religious norms and levels of socioeconomic development, may be biased due to the complex range of influences affecting the cultural group. In our study, the Europeans (Poles and Croatians) could be characterized as more individualistic, wealthy and mainly Christian (Roman Catholic). The Iranian sample, in turn, represented more collectivistic, less wealthy and mainly Islamic culture. On one hand, individualistic cultures tend to endorse expressions of emotion, whereas collectivistic cultures encourage control of expressions of affect to maintain group harmony. On the other hand, both Catholicism and Islam promote mental well-being over hedonism (Joshanloo, 2013). The affective ratings given to NAPS pictures by the European and Iranian samples may therefore be interpreted in terms of more general cultural differences between the groups. Detailed cross-cultural variations between the European and Iranian samples were observed, in the aforementioned MWW analysis, conducted for ratings of each of the images separately on each scale. However, no significant differences were observed between the ratings given to each picture category by European and Iranian samples. It might be that more radically distinct cultural groups would demonstrate greater differences in terms of affective ratings and give greater support to the hypothesis that arousal is a culture-sensitive component of affective states.

### Study Limitations and Conclusions

The research reported in this study used subjective ratings only of affective visual stimuli. We suggest that further research should include physiological and neuroimaging methods, in order to investigate reliable correlates for valence, arousal, and basic emotions.

Our results also require further verification due to the small sample size used. We hypothesized that cultural differences would have no effect on the ratings of picture content categories in terms of affective dimensions and basic emotions. However, although we did not find statistically significant differences in this respect between the two groups, it is unclear whether this corresponds to a true null hypothesis or simply a failure to reject it (false negative). Thus, the results in this paper should be treated with caution and call for confirmation with more experimental data, perhaps gathered from larger samples. Older people or children could be included, which might also reveal greater cross-cultural differences. Older generations may be more affected by geopolitical factors, such as wars and attacks, while children can demonstrate wider variations in affective ratings, reflecting underdeveloped emotion regulation (Tahmouresi et al., 2014).

The subjective behavioral ratings presented here were collected from both Europeans and Iranians in English, which was the second language of both groups. Despite the participants’ proficiency in English, using a non-native language in affective studies may influence levels of anxiety and therefore of arousal (Caldwell-Harris and Ayçiçegi-Dinn, 2009). We suggest that future studies should combine linguistic and affective variables to investigate the mediating effect of using non-native language on subjective affective ratings.

Finally, further research would benefit from collecting affecting ratings not only for NAPS, but also for another standardized dataset of affective pictures, such as IAPS. This would provide a broader context and contribute to better understanding of the NAPS results.

Overall, our results show the usefulness of NAPS as a tool in affective research. The correlation we observed between the ratings for affective dimensions has been proven in many studies to be a crucial characteristic for affective stimuli (Lang et al., 2008; Marchewka et al., 2014). These effects were not found to be culture dependent in the samples of Europeans and Iranians. However, we suggest that with larger samples they may appear. Our results furthermore highlight the importance of cross-cultural adaptations, in particular with regard to the content of the stimuli used in affective studies.

## Author Contributions

equal contribution: MR and AMo. Conception: AMa and AMo. Experimental design: AMa and AMo. Data acquisition: AMo, ŁŻ, and MH. Data analysis: MR, JM, MW, and ŁŻ. Interpretation: MR and AMa. Writing manuscript: MR. Corrections: AMa, KJ, and MR.

## Conflict of Interest Statement

The authors declare that the research was conducted in the absence of any commercial or financial relationships that could be construed as a potential conflict of interest.
