# Supplementary material for: Nencki Affective Picture System: Cross-Cultural Study in Europe and Iran
Source: Front Psychol. 2017 Mar 3;8:274. doi: 10.3389/fpsyg.2017.00274 (PMC5334317; doi:10.3389/fpsyg.2017.00274)
Supplement: Supplementary file 3 [file Data_Sheet_3.docx]

Appendix 1

*The results of ANOVA analyses and post-hoc pairwise comparisons for each affective dimension and basic emotion, with picture content category and cultural groups as factors.*

| **Affective dimension/ basic emotion** | **Main effect of picture content category** | **Significant post-hoc Bonferroni pairwise comparisons** | **Main effect of cultural group** | **Interaction of content and cultural group** |
| --- | --- | --- | --- | --- |
| Arousal | *F*(4, 308) = 93.696, *p* <.001, η^2^ = .549 | 1-2 (p < .001) | *F*(1, 77) = .003, *p* <.956, η^2^ = .000 | *F*(4, 308) = 2.201, *p* <.069, η^2^ = .028 |
|  |  | 1-3 (p < .001) |  |  |
|  |  | 1-4 (p < .001) |  |  |
|  |  | 2-3 (p < .001) |  |  |
|  |  | 2-4 (p < .001) |  |  |
|  |  | 3-4 (p < .015) |  |  |
|  |  | 5-1 (p < .001) |  |  |
|  |  | 5-2 (p < .001) |  |  |
|  |  | 5-3 (p < .001) |  |  |
|  |  | 5-4 (p < .001) |  |  |
| Valence | *F*(4, 308) = 170.373, *p* <.001, η^2^ = .689 | 1-4 (p < .509) | *F*(1, 77) = 3.087, *p* <.083, η^2^ = .039 | *F*(4, 308) = 1.689, *p* <.177, η^2^ = .021 |
|  |  | 1-5 (p. < .001) |  |  |
|  |  | 2-4 (p < .003) |  |  |
|  |  | 2-5 (p < .001) |  |  |
|  |  | 3-1 (p < .001) |  |  |
|  |  | 3-2 (p < .001) |  |  |
|  |  | 3-4 (p < .001) |  |  |
|  |  | 3-5 (p < .001) |  |  |
| Anger | *F*(4, 308) = 25.095, *p* <.001, η^2^ = .246 | 1-2 (p < .008) | *F*(1, 77) = .002, *p* <.967, η^2^ = .000 | *F*(4, 308) = .557, *p* <.622, η^2^ = .007 |
|  |  | 1-3 (p < .001) |  |  |
|  |  | 1-4 (p. < .001) |  |  |
|  |  | 2-3 (p < .001) |  |  |
|  |  | 2-4 (p < .001) |  |  |
|  |  | 3-4 (p < .029) |  |  |
|  |  | 5-2 (p < .001) |  |  |
|  |  | 5-3 (p < .001) |  |  |
|  |  | 5-4 (p < .001) |  |  |
| Disgust | *F*(4, 308) = 93.059, *p* <.001, η^2^ = .547 | 1-2 (p < .001) | *F*(1, 77) = .709, *p* <.402, η^2^ = .009 | *F*(4, 308) = 2.590, *p* <.071, η^2^ = .033 |
|  |  | 1-3 (p < .001) |  |  |
|  |  | 1-4 (p. < .001) |  |  |
|  |  | 2-3 (p < .001) |  |  |
|  |  | 4-2 (p < .001) |  |  |
|  |  | 4-3 (p < .001) |  |  |
|  |  | 5-1 (p. < .026) |  |  |
|  |  | 5-2 (p < .001) |  |  |
|  |  | 5-3 (p < .001) |  |  |
|  |  | 5-4 (p < .001) |  |  |
| Fear | *F*(4, 308) = 82.164, *p* <.001, η^2^ = .516 | 1-2 (p < .001) | *F*(1, 77) = .022, *p* <.882, η^2^ = .000 | *F*(4, 308) = 2.123, *p* <.122, η^2^ = .027 |
|  |  | 1-3 (p < .001) |  |  |
|  |  | 1-4 (p. < .001) |  |  |
|  |  | 2-3 (p < .001) |  |  |
|  |  | 2-4 (p < .001) |  |  |
|  |  | 4-3 (p < .001) |  |  |
|  |  | 5-2 (p < .001) |  |  |
|  |  | 5-3 (p < .001) |  |  |
|  |  | 5-4 (p < .001) |  |  |
| Happiness | *F*(4, 308) = 141.607, *p* <.001, η^2^ = .648 | 1-4 (p. < .001) | *F*(1, 77) = .022, *p* <.882, η^2^ = .000 | *F*(4, 308) = 2.561, *p* <.114, η^2^ = .032 |
|  |  | 1-5 (p. < .001) |  |  |
|  |  | 2-4 (p < .001) |  |  |
|  |  | 2-5 (p < .001) |  |  |
|  |  | 3-1 (p < .001) |  |  |
|  |  | 3-2 (p < .001) |  |  |
|  |  | 3-4 (p < .001) |  |  |
|  |  | 3-5 (p < .001) |  |  |
| Sadness | *F*(4, 308) = 160.355, *p* <.001, η^2^ = .676 | 1-3 (p. < .001) | *F*(1, 77) = 2.557, *p* <.065, η^2^ = .032 | *F*(4, 308) = .435, *p* <.512, η^2^ = .006 |
|  |  | 1-4 (p. < .001) |  |  |
|  |  | 2-1 (p < .001) |  |  |
|  |  | 2-3 (p < .001) |  |  |
|  |  | 2-4 (p < .001) |  |  |
|  |  | 4-3 (p < .001) |  |  |
|  |  | 5-1 (p < .001) |  |  |
|  |  | 5-2 (p < .001) |  |  |
|  |  | 5-3 (p < .001) |  |  |
|  |  | 5-4 (p < .001) |  |  |
| Surprise | *F*(4, 308) = 41.011, *p* <.001, η^2^ = .348 | 1-2 (p. < .001) | *F*(1, 77) = .505, *p* <.479, η^2^ = .007 | *F*(4, 308) = 1.108, *p* <.341, η^2^ = .014 |
|  |  | 1-3 (p. < .001) |  |  |
|  |  | 1-4 (p. < .001) |  |  |
|  |  | 4-3 (p < .040) |  |  |
|  |  | 5-2 (p < .001) |  |  |
|  |  | 5-3 (p < .001) |  |  |
|  |  | 5-4 (p < .001) |  |  |

*1- animal, 2 - face, 3 - landscape, 4 - object, 5 - people*
